# AMP-activated protein kinase reduces inflammatory responses and cellular senescence in pulmonary emphysema

**DOI:** 10.18632/oncotarget.15116

**Published:** 2017-02-06

**Authors:** Xiao-Yu Cheng, Yang-Yang Li, Cheng Huang, Jun Li, Hong-Wei Yao

**Affiliations:** ^1^ School of Pharmacy, Anhui Medical University, Hefei, The People's Republic of China

**Keywords:** cigarette smoke, AMPK, SIRT3, mitochondrial dysfunction, COPD

## Abstract

Current drug therapy fails to reduce lung destruction of chronic obstructive pulmonary disease (COPD). AMP-activated protein kinase (AMPK) has emerged as an important integrator of signals that control energy balance and lipid metabolism. However, there are no studies regarding the role of AMPK in reducing inflammatory responses and cellular senescence during the development of emphysema. Therefore, we hypothesize that AMPK reduces inflammatroy responses, senescence, and lung injury. To test this hypothesis, human bronchial epithelial cells (BEAS-2B) and small airway epithelial cells (SAECs) were treated with cigarette smoke extract (CSE) in the presence of a specific AMPK activator (AICAR, 1 mM) and inhibitor (Compound C, 5 μM). Elastase injection was performed to induce mouse emphysema, and these mice were treated with a specific AMPK activator metformin as well as Compound C. AICAR reduced, whereas Compound C increased CSE-induced increase in IL-8 and IL-6 release and expression of genes involved in cellular senescence. Knockdown of AMPKα1/α2 increased expression of pro-senescent genes (e.g., p16, p21, and p66shc) in BEAS-2B cells. Prophylactic administration of an AMPK activator metformin (50 and 250 mg/kg) reduced while Compound C (4 and 20 mg/kg) aggravated elastase-induced airspace enlargement, inflammatory responses and cellular senescence in mice. This is in agreement with therapeutic effect of metformin (50 mg/kg) on airspace enlargement. Furthermore, metformin prophylactically protected against but Compound C further reduced mitochondrial proteins SOD2 and SIRT3 in emphysematous lungs. In conclusion, AMPK reduces abnormal inflammatory responses and cellular senescence, which implicates as a potential therapeutic target for COPD/emphysema.

## INTRODUCTION

Cigarette smoke is a major risk factor for the development of chronic obstructive pulmonary disease (COPD). Current drug therapy fails to reduce the relentless progression of COPD, which may be due to lack of understanding of the mechanisms underlying lung destructive processes of this disease. Cellular senesence interacts with microenvironmental inflammation, which plays a pivotal role in the pathogenesis of COPD [1–3]. Nevertheless, the mechanisms underlying cellular senescence and inflammatory responses during the development of COPD are not well understood.

The AMP-activated protein kinase (AMPK) is a conserved kinase, which contains three subunits α, β, and γ. The phosporylation of Thr-172 on the α subunit is critical for AMPK activation in response to an increase in AMP concentation. AMPK is an important integrator of signals that control energy balance and metabolism [4]. Recent studies have shown that AMPK participates in regulation of cellular senescence and premature aging [5, 6]. However, there are no studies regarding the regulation of AMPK in lung inflammatory responses and cellular senescence during the development of COPD/emphysema. Therefore, we hypothesize that AMPK reduces inflammatroy responses and senescence during the devlopment of pulmonary emphysema. To test this hypothesis, we utilized human bronchial epithelial cells (BEAS-2B) and small airway epithelial cells (SAECs) treated with cigarette smoke extract (CSE) as well as a mouse model of elastase-induced pulmonary emphysema.

## RESULTS

### Effect of AMPK on inflammatory mediator release in human lung epithelial cells

**Figure 1:**
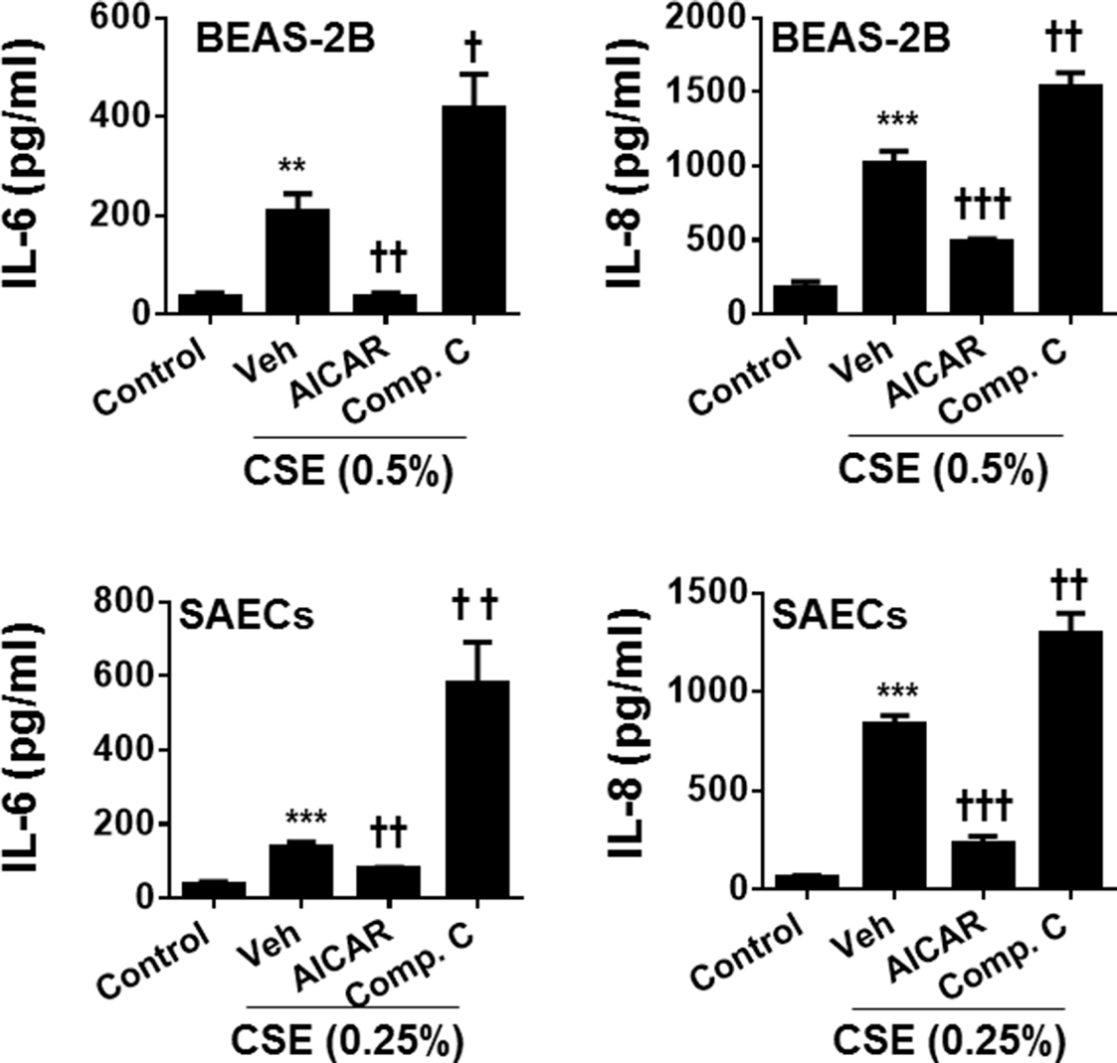
Effect of AMPK on CSE-induced release of pro-inflammatory mediators in human lung epithelial cells Both BEAS-2B and SAECs were treated with AICAR (1 mM) or Compound C (5 μM) for 24 h in the presence or absence of CSE (0.25% and 0.5%) treatment. Culture supernatants were collected for the measurement of IL-6 and IL-8 by ELISA. Data are expressed as the mean ± SEM. *N* = 4–5. ^*^P < 0.01, ^**^*P < 0.001, vs. control; ^†^*P* < 0.05, ^††^*P* < 0.01, ^†††^*P* < 0.001, vs. CSE-Veh group.

Cigarette smoke is known to cause inflammatory responses, which are important features during the development of COPD/emphysema. To determine the role of AMPK in reducing inflammatory responses, both BEAS-2B and SAEC cells were treated with a specific AMPK activator (AICAR, 1 mM) or inhibitor (Compound C, 5 μM) during CSE treatment for 24 hours. As shown in Figure 1, CSE treatment caused a significant release of pro-inflammatory mediators IL-6 and IL-8 in both BEAS-2B and SAECs. Treatment with an AMPK activator AICAR reduced, whereas Compound C increased CSE-induced release in pro-inflammatory mediators IL-6 and IL-8. These results demonstrate that AMPK attenuates cigarette smoke-induced inflammatory responses in human lung epithelial cells.

### AMPK reduced the expression of genes involved in cellular senescence in human lung epithelial cells

**Figure 2:**
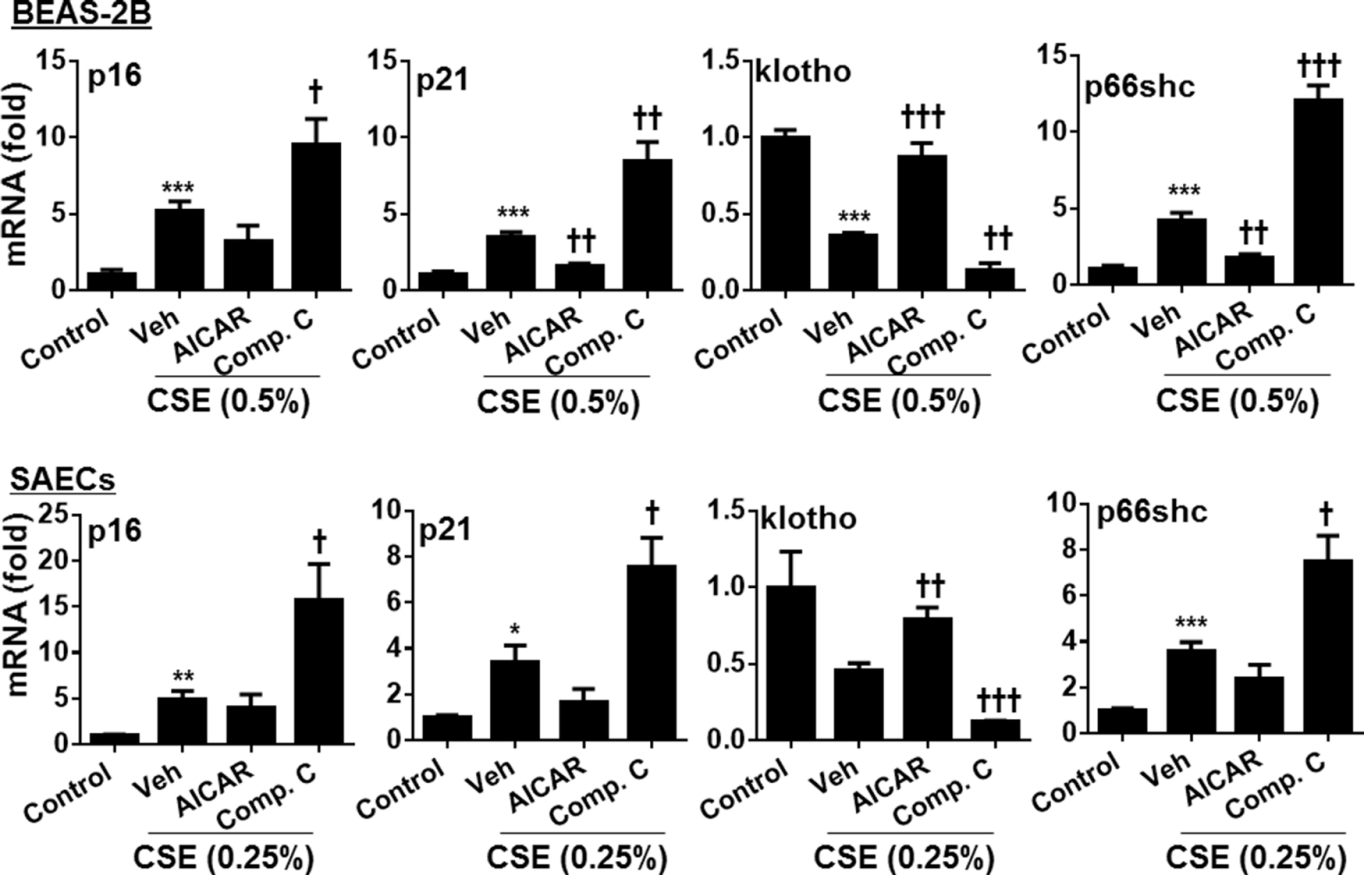
Effect of AMPK on expression of p16, p21, klotho and p66shc genes in human lung epithelial cells treated with CSE Both BEAS-2B and SAECs were treated with AICAR (1 mM) or Compound C (5 μM) for 24 h in the presence or absence of CSE (0.25% and 0.5%) treatment. Cell lysates were used for detecting the expression of p16, p21, klotho and p66shc by real-time PCR. Data are expressed as the mean ± SEM. *N* = 4–5. *P < 0.05, ^*^P < 0.01, ^**^*P < 0.001, vs. control; ^†^*P* < 0.05, ^††^*P* < 0.01, ^†††^*P* < 0.001, vs. CSE-Veh group.

Senescent cells are not quiescent cells, which show increased inflammatory phenotype in response to stress. Therefore, we hypothesize that AMPK ameliorates senescent responses in human lung epithelial cells exposed to cigarette smoke. As shown in Figure 2, CSE treatment increased the expression of p16, p21 and p66shc but reduced klotho gene expression. AICAR treatment reduced the expression of p16, p21, and p66shc, but augmented klotho gene expression in both BEAS-2B and SAEC cells treated with CSE. In contrast, Compound C treatment further enhanced CSE-induced expression of p16, p21, and p66shc, whereas klotho gene expression was reduced by Compound C in human lung epithelial cells. These results indicate that AMPK reduces cigarette smoke-induced senescence in lung epithelial cells.

### AMPKα1/α2 knockdown increased expression gene involved in cellular senescence

**Figure 3:**
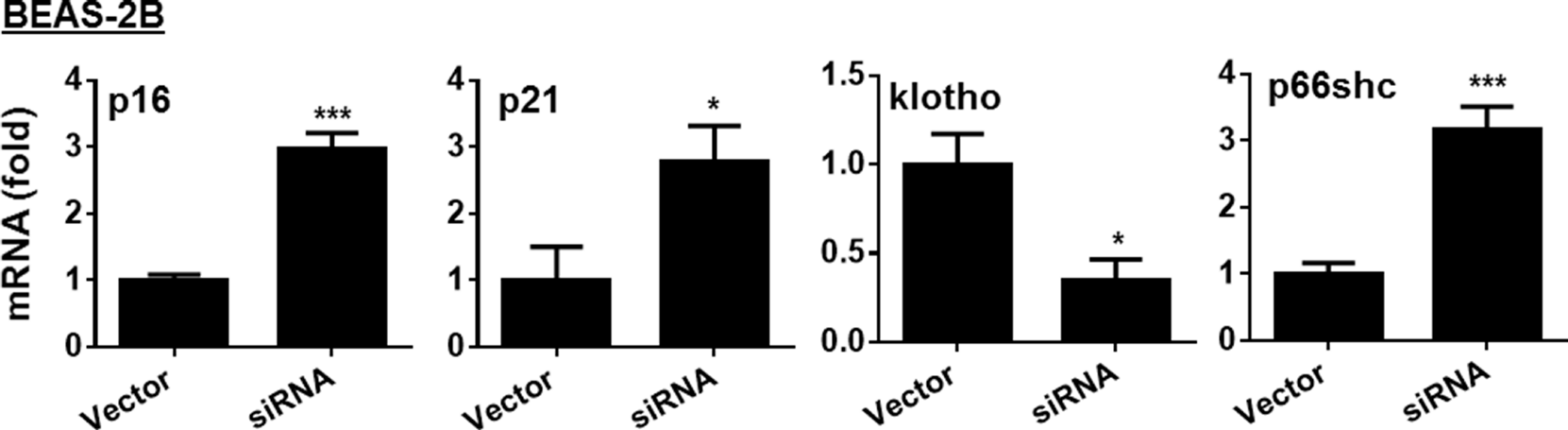
Effect of AMPK siRNA on expression of p16, p21, klotho and p66shc genes in human bronchial epithelial cells Human bronchial epithelial cells (BEAS-2B) were transfected with AMPKα1/α2 siRNA for 24 h, and the expression of p16, p21, klotho and p66shc was determined by real-time PCR. Data are expressed as the mean ± SEM. *N* = 4–5. *P < 0.05, ^**^*P < 0.001, vs. Vector.

To further determine role of AMPK in regulating cellular senescence, we transfected BEAS-2B cells with AMPKα1/α2 siRNA. As shown in Figure 3, transfection of AMPKα1/α2 siRNA increased the mRNA of p16, p21 and p66shc, but reduced klotho gene expression in BEAS-2B cells. Altogether, AMPK reduces expression of genes associated with cellular senescence.

### AMPK prophylactically and therapeutically attenuated elastase-induced airspace enlargement

**Figure 4:**
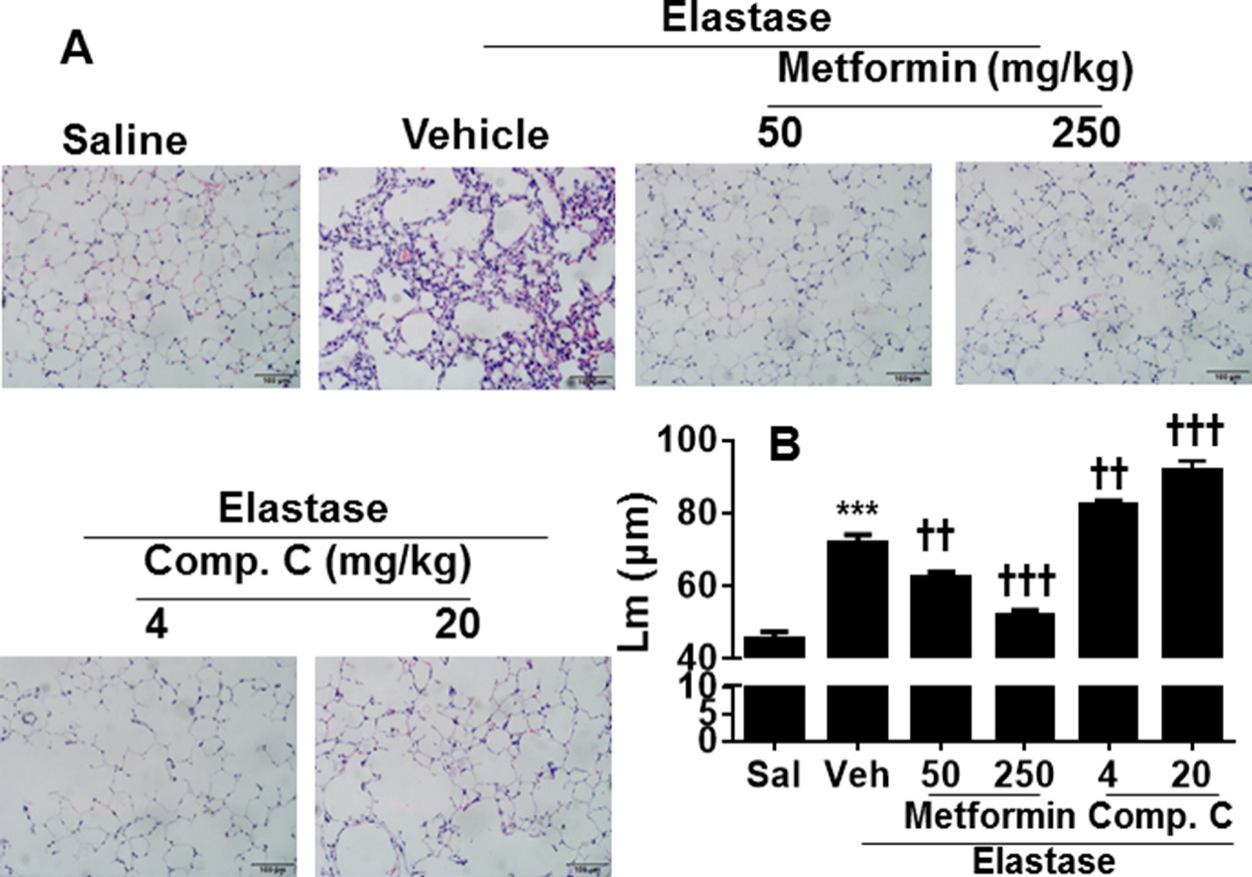
AMPK prophylactically attenuated elastase-induced airspace enlargement in mice C57BL/6J mice were intracheally injected with elastase, and metformin (50 and 250 mg/kg) as well as Compound C (4 and 20 mg/kg) was administered into these mice through oral gavage. Mean linear intercept (Lm) was measured in H&E stained lung tissues. Bar size: 100 μm. Data are expressed as the mean ± SEM. *N* = 4–5. ^**^*P < 0.001, vs. saline; ^††^*P* < 0.01, ^†††^*P* < 0.001, vs. elastase-Veh group. and

**Figure 5:**
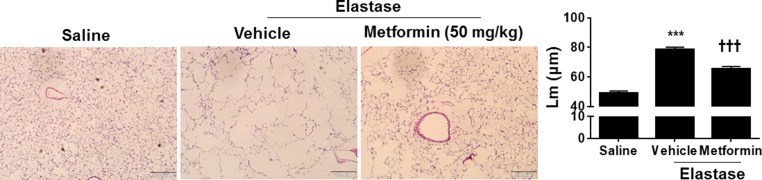
Metformin therapeutically attenuated elastase-induced airspace enlargement in mice C57BL/6J mice were intracheally injected with elastase, and after 3 weeks of elastase instillation metformin (50 mg/kg) was administered daily into these mice through oral gavage for 1 week. Mean linear intercept (Lm) was measured in H&E stained lung tissues. Bar size: 200 μm. Data are expressed as the mean ± SEM. *N* = 3–4. ^**^*P < 0.001, vs. saline; ^†††^*P* < 0.001, vs. elastase-Veh group.

To further extrapolate the *in vitro* findings into *in vivo* animal model, we established a mouse model of pulmonary emphysema, as described previously [7]. As shown in Figure 4, a significant increase in mean linear intercept (Lm) was observed in mice injected with elastase. Prophylactic administration of a specific AMPK activator metformin (50 and 250 mg/kg) apparently attenuated elastase-induced airspace enlargement, with higher efficacy at dose of 250 mg/kg (Figure 4). In contrast, treatment with a specific AMPK inhibitor Compound C (4 and 20 mg/kg) significantly augmented elastase-induced increase in Lm. Furthermore, we started to administer metformin (50 mg/kg, daily, 1 week) to mice after 3 weeks of intratracheal elastase instillation. We found that therapeutic administration of metformin significantly reduced airspace enlargement (Figure 5). These findings implicate that AMPK activation is beneficial to intervene with development of emphysema via prophylactic and therapeutic actions

### AMPK reduced inflammatory responses and senescence in mice with emphysema

**Figure 6:**
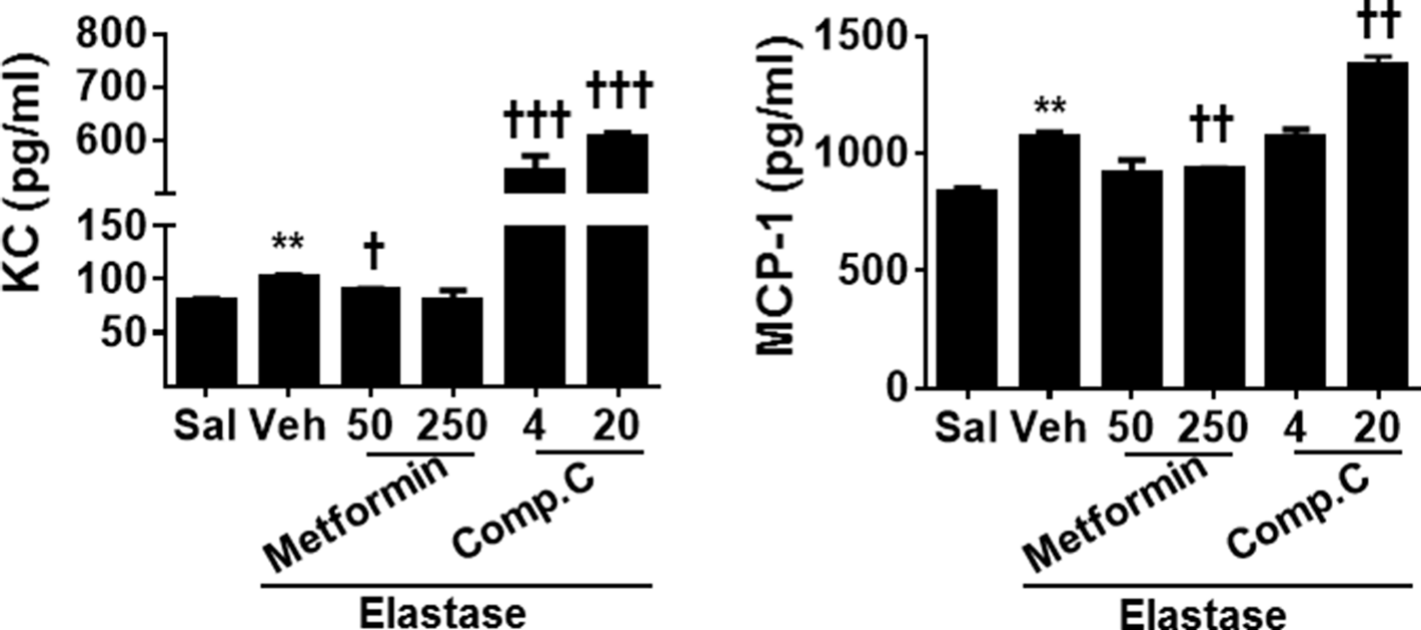
AMPK prophylactically reduced pro-inflammatory cytokine release in BAL fluid in emphysematous mice C57BL/6J mice were intracheally injected with elastase, and metformin (50 and 250 mg/kg) as well as Compound C (4 and 20 mg/kg) was administered into these mice through oral gavage. BAL fluid was collected, and levels of KC and MCP-1 were measured by ELISA. Data are expressed as the mean ± SEM. *N* = 4–5. ^*^P < 0.001, vs. saline; ^†^*P* < 0.05, ^††^*P* < 0.01, ^†††^*P* < 0.001, vs. elastase-Veh group.

To determine whether AMPK modulates inflammatory responses and cell senescence *in vivo*, we first measured the levels of cytokines KC and MCP-1 by ELISA in bronchoalveolar lavage (BAL) fluid of mice with emphysema. Levels of KC and MCP-1 were significantly increased in BAL fluid of emphysematous mice as compared to control saline group (Figure 6). Prophylactic treatment with metformin attenuated, whereas Compound C administration further increased the levels of KC and MCP-1 in BAL fluid in a mouse model of COPD induced by elastase (Figure 6).

**Figure 7:**
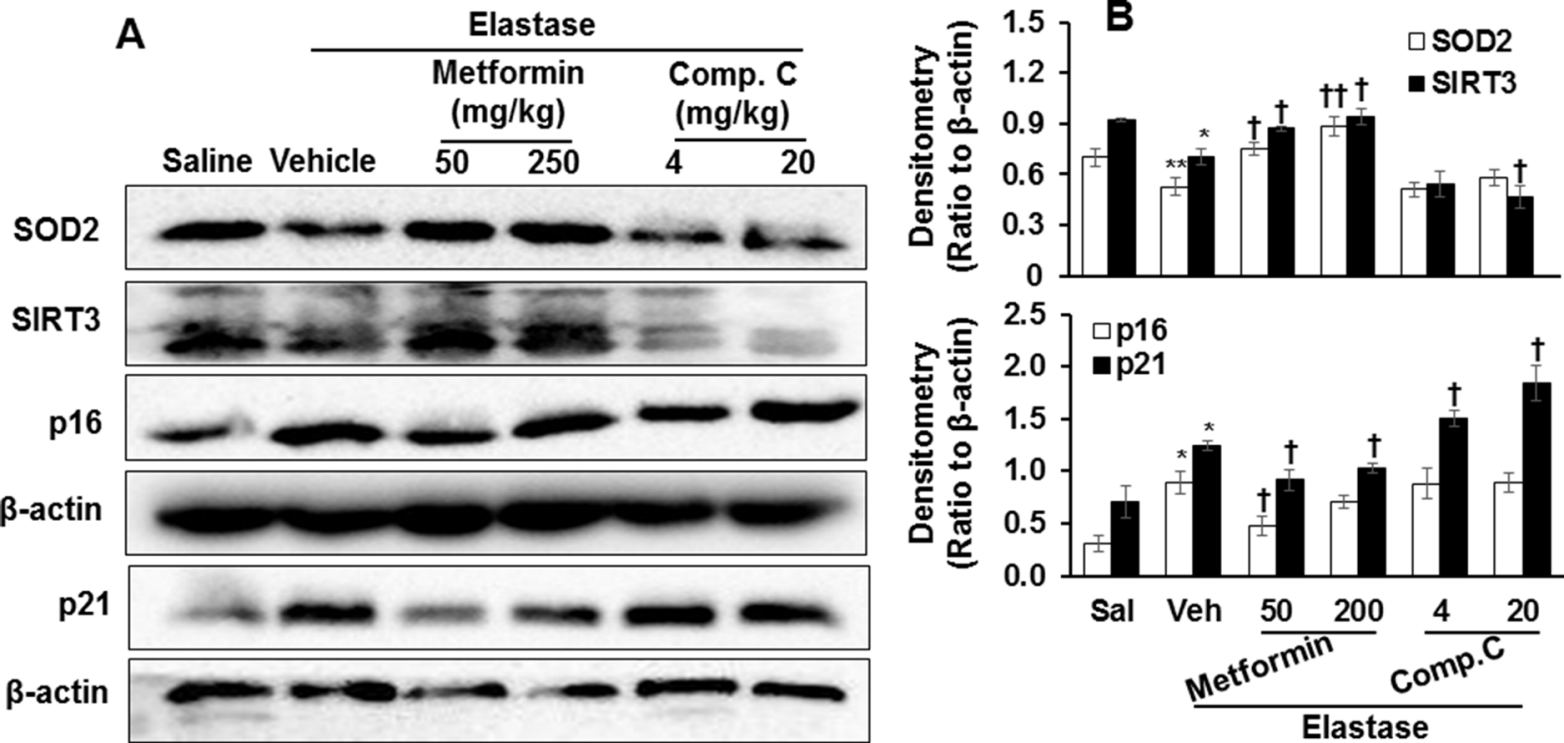
Prophylactically effect of AMPK on levels of p16, p21, SOD2, and SIRT3 proteins in mouse lungs with emphysema C57BL/6J mice were intracheally injected with elastase, and metformin (50 and 250 mg/kg) as well as Compound C (4 and 20 mg/kg) was administered into these mice through oral gavage. (**A**) Protein levels of p16, p21, SOD2, and SIRT3 proteins were measured by Western blot, and β-actin was used as loading control. (**B**) Densitometry of bands were normalized to β-actin, which were expressed as the mean ± SEM. *N* = 4–5. *P < 0.05, ^*^P < 0.01, vs. saline; ^†^*P* < 0.05, ^††^*P* < 0.01, vs. elastase-Veh group.

**Figure 8:**
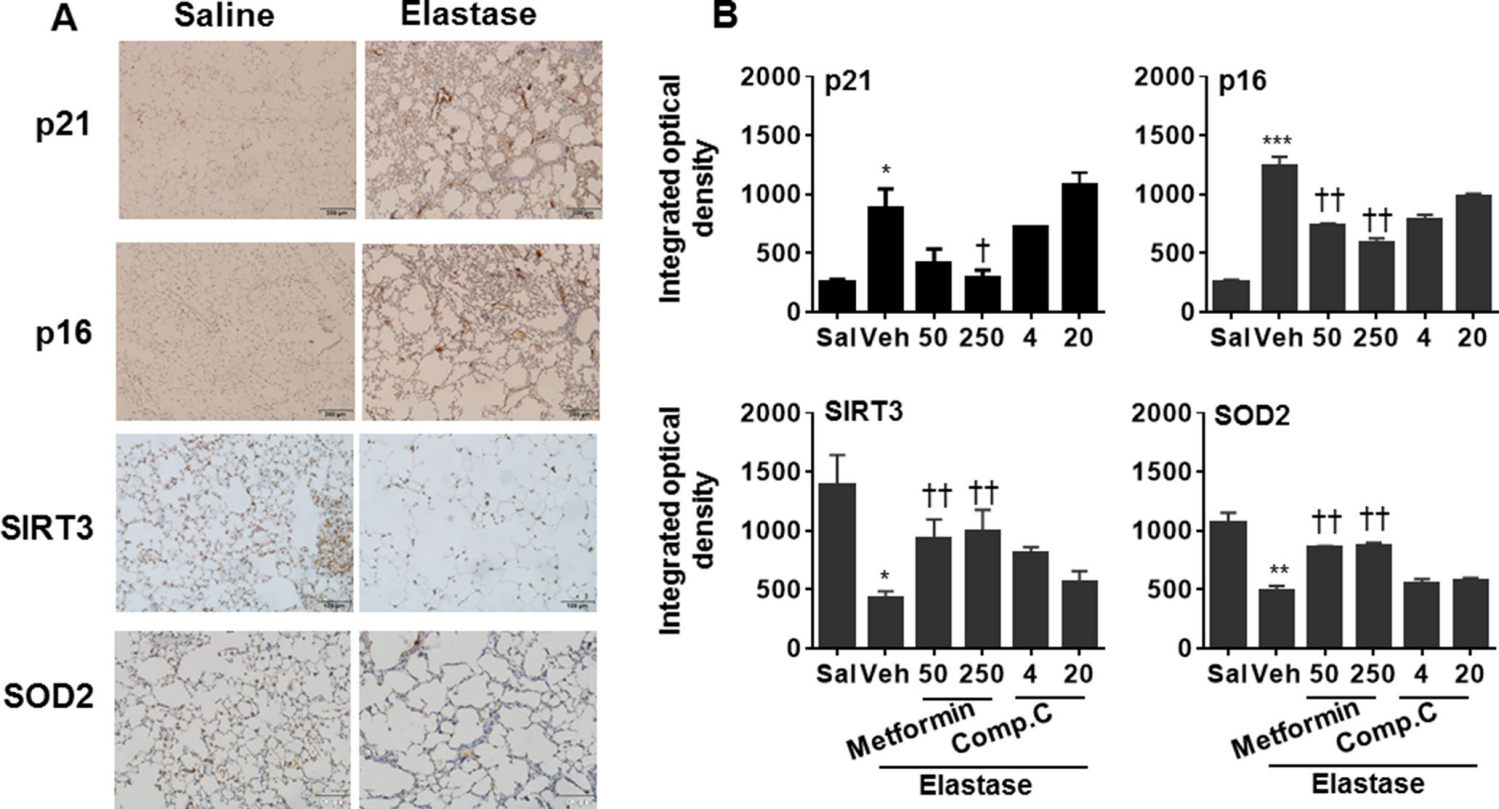
Prophylactically effect of AMPK on abundance of p16, p21, SOD2, and SIRT3 proteins in mouse lungs with emphysema C57BL/6J mice were intracheally injected with elastase, and metformin (50 and 250 mg/kg) as well as Compound C (4 and 20 mg/kg) was administered into these mice through oral gavage. (**A**) Representative images of p16, p21, SOD2, and SIRT3 protein abundance in saline and elastase groups by immunohistochemistry. (**B**) Semi-quantitative analysis was performed using the Image pro-plus 6.0 software. *P < 0.05, ^*^P < 0.01, ^**^*P < 0.001, vs. saline; ^†^*P* < 0.05, ^††^*P* < 0.01, vs. elastase-Veh group.

Inflammation and cellular senescence are intertwined in the pathogenesis of COPD [8, 9]. Hence, we also determine the expression of p16 and p21, markers of cellular senescence, in lung tissues using Western blot and immunohistochemistry. As expected, intratracheal inject of elastase significantly caused cellular senescence as reflected by an increase in p16 and p21 proteins in mouse lungs, and these effects were reduced by prophylactic treatment of metformin (Figure 7 and Figure 8). In contrast, Compound C further enhanced the abundance of p16 and p21 proteins in emphysematous mice induced by elastase. All together, these findings suggest that AMPK protects against emphysema by reducing inflammatory responses and cellular senescence.

### AMPK increased the abundance of SOD2 and Sirtuin3 (SIRT3) proteins in mice with emphysema

Mitochondrial function plays an important role in regulating inflammatory responses and cellular senescence [10–12]. Therefore, we measured the abundance of mitochondrial proteins SOD2 and SIRT3 in emphysematous lungs treated with specific AMPK activator and inhibitor. In response to elastase, the abundance of SOD2 and SIRT3 proteins was significantly reduced as compared to saline control (Figures 7 and 8). The expression of SOD2 and SIRT3 proteins was augmented by prophylactic treatment of metformin, whereas Compound C further caused reduction of SOD2 and SIRT3 proteins in emphysematous lungs (Figures 7 and 8). These results indicate that the protective effects of AMPK on emphysema are associated with the up-regulation of mitochondrial proteins SOD2 and SIRT3.

## DISCUSSION

The inflammatory responses and cellular senescence are closely entwined during the development of COPD/emphysema [1, 13]. The mechanisms for lung cellular senescence during the progression of COPD are not well understood. Here we found that AMPK reduced cigarette smoke-induced lung cellular senescence and inflammatory responses *in vitro* using specific pharmacological activator and inhibitor as well as genetic knockdown. Furthermore, treatment with a specific AMPK activator metformin significantly attenuated whereas an AMPK inhibitor Compound C further augmented elastase-induced airspace enlargement in mice. Altogether, AMPK reduces lung cellular senescence and inflammatory responses, thereby protecting against the development of emphysema.

Cellular senescence is a status where cells lose the power to divide and grow. This is a protective mechanism to avoid further DNA damage and mutation in response to environmental and oxidative stress [14]. Senescent cells are not metabolically quite. In contrast, these cells are reprogrammed to prone to release inflammatory mediators and matrix metalloproteinases, which further cause and reinforce senescence and damage in neighbor cells [15]. Recent studies highlight the importance of cellular senescence in the pathogenesis of chronic lung diseases including COPD and pulmonary fibrosis [16–19]. We showed here that cigarette smoke *in vitro* and elastase *in vivo* augmented the expression of corresponding p21 and p16 genes and proteins, which are key p53-p21 and p16-pRb pathways for initiating and propagating cellular senescence [20]. It is interestingly to note that elastase treatment (50 mU/ml, 24 h) did not affect the mRNA levels of p21 and p16 in BEAS-2B cells (data not shown). This suggests that the mechanisms underlying elastase-induced cellular senescence are different from cigarette smoke, which needs further investigation. Klotho gene encodes a single-pass transmembrane protein that is expressed primarily in renal tubules [21, 22]. Klotho functions as an anti-aging factor, and suppresses stress-induced cellular senescence associated with inflammatory responses [23]. There are several reports showing the reduction of klotho in lung inflammatory and epithelial cells of COPD patients, which contributes to inflammation and oxidative stress [24, 25]. Importantly, klotho deficient mice exhibit the phenotype of emphysema [26, 27]. In agreement with these findings, cigarette smoke exposure reduced the expression of klotho gene in both human bronchial epithelial cells and small airway epithelial cells in the present study. Our findings further demonstrate the cellular senescence-associated inflammatory phenotype as one of the pathobiological mechanisms for the progression of COPD/emphysema, as the levels of pro-inflammatory cytokines (i.e., IL-6, IL-8, KC and MCP-1) were concurrently increased in CSE-treated human lung epithelial cells *in vitro* and elastase-exposed mouse lungs *in vivo*. Although elastase induces lung cellular senescence, inflammatory responses and oxidative stress which are intertwined to cause emphysema [18, 28, 29], the long-term exposure of cigarette smoke to lung epithelial cells is required to determine cellular senescence phenotype and its association with re-epithelialization.

A p66shc is a 66 kDa proto-oncogene Src homologous-collagen homologue (Shc) adaptor protein, which regulates intracellular oxidant levels, and is recently identified as a longevity protein in mammals [30]. The p66shc mainly localizes in mitochondria, and regulates mitochondrial reactive oxygen species (ROS)-mediated signals [31]. Recent reports demonstrated that mitochondria ROS and turnover play an important role in cellular senescence and apoptosis during the development of COPD [16, 17, 32]. Furthermore, p66shc knockout mice developed respiratory bronchiolitis with fibrosis but not emphysema in response to cigarette smoke exposure [33]. This is corroborated by our findings that mitochondrial antioxidant SOD2 and histone deacetylase SIRT3 were reduced in mouse lungs with emphysema. SOD2 neutralizes mitochondrial ROS [34, 35], while SIRT3 regulates mitochondrial biogenesis and dynamics [36, 37]. Therefore, the alterations of p66shc, SOD2 and SIRT3 may link mitochondrial ROS, biogenesis and dynamics to cellular senescence during the development of COPD/emphysema.

AMPK is a serine and threonine protein kinase, which modulates cellular energy homeostasis metabolism [4]. It has been labeled as an anti-aging and anti-inflammatory molecule [5, 6]. Our preliminary findings show the reduction of AMPKα1 phosporylation at Thr-172 in lungs of COPD pateints as compared to nonsmokers (data not shown). This propels us to study the role of AMPK in regulating lung inflammatory responses and senscence. We for the first time found that an AMPK activator AICAR reduced, whereas both AMPK inhibition by Compound C and siRNA transfection enhanced cigarette smoke-induced expression of genes involved in cellular senescence. This may be one the mechanisms for anti-inflammatory action (i.e., reduction of IL-6, IL-8, KC and MCP-1) of AMPK in response to cigarette smoke and elastase exposures. This is corroborated by the studies showing that AMPK inhibits lung inflammatory responses to cigarette smoke [38, 39]. A recent report demonstrates that AMPK levels were reduced in the aortas of klotho knockout mice [40]. It remains unknown whether klotho affects AMPK activation during the development of emphysema.

Mitochondrial dysfunction plays an important role in initiating inflammatory responses and cellular senescence [10]. Indeeed, we found that AMPK negative regulated p66shc but promoted the expression of SOD2 and SIRT3, and all molecules are mainly distributed in mitochondria. Both SOD2 and SIRT3 negatively regulates stress-induced cellular senescence [41–43]. Therefore, the inhibitory effects of AMPK againts inflammatory responses and cellluar senescence may be due to its protection against mitochondrial dysfunction. Further study is required for determining the mechanisms by which AMPK regulates mitochondrial function and dysnamics.

A recent study has shown that genetic knockout of AMPKα1 enhances lung inflammatroy responses and emphysema in mice exposed to cigarette smoking and poly(I:C) [38]. We confirmed this finding using an elastase-induced emphysema model as well as prophylactic and therapeutic treatments with a specific pharmacological AMPK activator metformin. The ongoing experiments are ongoing to determine the therapeutic effects of AMPK activators during the development of emphysema induced by long-term cigareette smoke exposure. Interestingly, there are two reports showing that cigarette smoke promotes AMPK phophorylation, and AMPK inhibiton reduced inflammatory responses in lung epithelial cells and macrophages [44, 45]. The descripancies among these studies are not clear, which may due to the differences of cells (inflammatory vs. structural cells; primary vs immortal cells), cigarette smoke concentrations and durations. It should be noted that both metformin and compound C have pleiotropic effects [46, 47], and AMPK knockout mice treated with these compounds would further consolidate the role of AMPK in the development of COPD/emphysema.

Although both cigarettt smoke and elastase induce pulmonary emphysema through proteolytic degradation of the lung matrix, the mechanisms of this process are likely very different [48]. Further study on these mechanisms would reveal how AMPK protects against lung inflammation and senescence, and lay a foundation to identify potential therapeutic targets for COPD treatment. In the preset study, we did not use the same stimulus (cigarette smoke or elastase) for both *in vitro* and *in vivo* studies. This is due to the facts that the cigarette smoke model does not reproduce the most severe aspects of the disease observed in humans, and requires 4–6 months of exposure [49]. In contrast, in the elastase-induced emphysema model, the disease develops rapidly and severely, which is relatively easy (one time injection) and cheap (do not need smoke machine/facility) to evaluate potential therapeutics and interventions for emphysema [50]. Hence, we employed an *in vivo* elastase (rather than cigarette smoke) model to determine the role of AMPK in protecting against pulmonary emphysema.

In conclusion, cigarette smoke exposure caused inflammatory responses and expression of genes involved in senescence in human bronchial epithelial cells and small airway epithelial cells. Treatment with a specific AMPK activator AICAR reduced, whereas Compound C enhanced cigarette smoke-induced inflammatory responses. Furthermore, administration of a specific AMPK activator metformin attenuated elastase-induced airspace enlargement, inflammatory responses and cellular senescence in mice. Therefore, AMPK is a promising therapeutic target for intervene with the progression of COPD/emphysema by regulating inflammatory responses and cellular senescence.

## MATERIALS AND METHODS

### Cell culture and treatments

Human bronchial epithelial cells BEAS-2B cells (Sigma) were cultured in Dulbecco's modified Eagle's medium (DMEM) supplemented with 10% heat-inactivated fetal bovine serum (FBS), 2 mM L-glutamine, penicillin (100 U/ml), and streptomycin (100 U/ml) in 5% CO_2_ and humid air at 37^°^C. Human small airway epithelial cells SAECs (Lonza) were maintained in Small Airway Epithelial Cell Growth Medium (SAGM; basal medium plus growth supplements, Lonza). Cells were used between passage 4 and 10. Cells were exposed to CSE (0.25% for SAECs and 0.5% for BEAS-2B based on their viability) for 24 hours in the presence or absence of 5-Aminoimidazole-4-carboxamide ribonucleotide (AICAR, 1 mM, Sigma) and Compound C (5 μM, Sigma).

### CSE preparation

A Kentucky research 3R4F cigarette was lit, and smoking was bubbled through 10 ml of cell culture medium at a speed of 1 min per cigarette using a negative pressure pump. This aqueous extract of smoking was considered as 10% CSE with an OD value of 1 at wavelength 320 nm, which was used for cell treatment after a filtration through a 0.2 mm sterile filter. The CSE was freshly prepared prior to cell treatment of each experiment to avoid the breakdown of substances in the extract and evaporation of volatile components.

### Quantitative real-time PCR

Total RNA was isolated using TRIzol (Invitrogen) according to the manufacturer's instructions. cDNA was generated using a Transcriptor first-strand cDNA synthesis kit (Qiagen), and the primers for p16, p21, klotho and p66shc were used for PCR amplification [51–54]. The primers were as follows: human *p16*, 5′-CCCAACG CACCGAATAGTTA-3′ (forward) and 5′-ACCAGCGT GTCCAGGAAG-3′ (reverse); p21, 5′-GGCCTGGACTG TTTTCTCTCG-3′ (forward) and 5′-GAGAAACGGGA ACCAGGACAC-3′ (reverse); klotho: 5′-TACCTGGTGG CGCACAACCT-3′ (forward) and 5′-TGTGGTCGGTC ATTCTTCGA-3′ (reverse); p66shc, 5′-CACTACCCTGT GCTCCTTCTTC-3′ (forward) and 5′-CGCCTCCACTC AGCTTGTT-3′(reverse); GAPDH, 5′-CGAGTCAACGGA TTTGGTGGTAT-3′ (forward) and 5′-AGCCTTCTCCAT GGTGAAGAC-3′ (reverse). Relative levels of specific mRNA were determined using the Thermo PIKOREAL 96 real-time PCR detection system with QIAGEN SYBR ® Green supermix (Valencia, CA, USA) according to the manufacturer's instructions. The GAPDH gene was used as an internal control for normalization.

### AMPKα1/α2 siRNA transfection

To reduce endogenous AMPKα expression, BEAS-2B cells were seeded onto a 6-well plate (1 × 10^6^/well) and transfected with human AMPKα1/α2 siRNA (Santa Cruz Technology) at 50 nM using the Lipofectamine 2000 (Invitrogen) for 24 hours according to the instructions. The following sequences were used: ATGATGTCAGA TGGTGAATTT for AMPKα1 and AATGGAATATGTGTC TGGAGG for AMPKα2 [55]. The ratio of siRNA over Lipofectamine 2000 in a well was 5 μl: 5 μl.

### Elastase-induced airspace enlargement and drug administration

C57BL/6J mice (2–3 months old, both sexes) were intratracheally administered with elastase after anesthesia with pentobarbital, as described previously [7]. A 100 μl of saline alone or saline containing 1 U of porcine pancreatic elastase (Sigma) was injected into tracheal using the MicrosSyringe with square tip [7, 56, 57]. Three weeks was allowed to develop pulmonary emphysema after elastase injection. During the third week after elastase instillation, metformin (50 and 250 mg/kg) or Compound C (4 and 20 mg/kg) was orally gavaged daily for 1 week to determine the prophylactic effect of AMPK on pulmonary emphysema. For therapeutic treatment, metformin (50 mg/kg) was orally administered daily for 1 week after 3 weeks of elastase injection when emphysema was established. The animal protocol was approved by the Ethics Committee of Anhui Medical University.

### BAL fluid collection

At the end of each experiment, pentobarbiturate (100 mg/kg) was intraperitoneally injected into mice following by exsanguination. Left lung was fixed for further histological analysis, and right lung was lavaged four times with PBS (0.6 ml each time). BAL fluid was collected and centrifuged at 350 g for 5 min at 4°C, and the supernatant of the lavage fluid was stored at −80°C for cytokine analysis.

### Cytokine measurement

After cell treatments, culture supernatants were collected through centrifugation, and then stored at −80°C until analysis. The concentrations of IL-6 and IL-8 were quantified by an enzyme-linked immunosorbant assay (ELISA) according to the manufacturer's instructions. The kits of human IL-6 and IL-8 were purchased from the R&D Systems (R&D Systems, CA). The OD values were read at 450 nm wavelength, and the real values of IL-6 and IL-8 were plotted based on their standard curves. Similarly, levels of mouse KC and MCP-1 in BAL fluid were measured by the ELISA using commercial kits (Elabscience Biotechnology Co, Ltd) according to the manufacturer's instructions.

### Hematoxylin and eosin stain for morphology

Left mouse lungs were inflated by 1% low melting agarose at a pressure of 25 cm H_2_O. The inflated lungs were fixed with 4% formalin, and then embedded in paraffin. Lung blocks were sectioned at thickness of 4 μm, and stained with hematoxylin and eosin. Alveolar size was estimated form the Lm of the airspace as described [58]. In brief, Lm was calculated for each sample based on 10 random fields observed at a magnification of ×200 using a cross-line.

### Immunohistochemistry

Lung blocks were deparaffinized in xylene and dehydrated in alcohol, and antigen retrieval was achieved by microwaving in citric saline for 15 min. Then sections were incubated with 3% H_2_O_2_ for 10 min to block endogenous peroxidase activity. After being blocked with 5% bovine serum albumin, the sample slides were incubated with primary antibody against SIRT3 (sc-99143, Santa Cruz Biotechnology), SOD2 (sc-133134, Santa Cruz Biotechnology), p21 (sc-397, Santa Cruz Biotechnology) and p16 (sc-377412, Santa Cruz Biotechnology) overnight at 4°C. After rinsing, the sections were incubated with corresponding secondary antibodies for an additional hour at room temperature. Antigenic sites were visualized by 3,3′-diaminobenzidine tetrahydrochloride (DAB) staining. The slides were counterstained with hematoxylin for 1 min, dehydrated and then observed using an Olympus microscope. Integrated optical density (IOD) of positive cells in lung sections (at least 3 random microscopic fields per lung section) was analyzed by the Image pro-plus 6.0 software.

### Western blot

Frozen right lung tissues were homogenized with RIPA buffer containing protease inhibitor. Aliquots of tissue lysates were used for protein analysis and then separated on 12% SDS–PAGE. Separate proteins were transferred onto the Immobilon-P transfer membranes 0.45 mm (Millipore). The membranes were blocked with 5% nonfat dry milk in Tris-buffered saline (TBS) with 0.05% Tween 20 (TBST). After blocking for 3 h, the membrane was incubated with primary antibodies (1:500 dilutions) in blocking buffer for 24h at 4°C. Primary antibodies against SOD2 (ab68155), SIRT3 (ab86671), p16 (ab51243) and p21 (ab109199) were purchased from Abcam. After washing with TBST for 3 times (10 min each), the membranes were incubated with secondary anti-rabbit antibody (1:10,000 dilutions in 5% skim milk) linked to HRP for 1 h at room temperature, and then the membranes were developed using ECL method (Millipore, Billerica, U.S.A). β-actin was used as a loading control. ImageJ densitometry software (Version 1.41; National Institutes of Health, Bethesda, MD) was used for the band quantification of Western blots.

### Statistical analysis

Statistical analyses were performed by SPSS 19.0. The results were expressed as mean ± SEM. The statistical significance of the differences between groups was evaluated by using one-way ANOVA followed by Student's *t*-test. Statistical significance was considered existing when *P* < 0.05.
